# The 4-(dimethylaminoalkyl)piperazine inhibitors of α-glucosidase: allosteric enzyme inhibition and identification of interacting chemical groups

**DOI:** 10.55730/1300-0527.3453

**Published:** 2022-05-20

**Authors:** Usman GHANI, Sajda ASHRAF, Zaheer UL HAQ, Zafer Asım KAPLANCIKLI, Fatih DEMİRCİ, Yusuf ÖZKAY, Sibtain AFZAL

**Affiliations:** 1,* Clinical Biochemistry Unit, Department of Pathology, College of Medicine, King Saud University, Riyadh, Saudi Arabia; 2 Dr. Panjwani Center for Molecular Medicine & Drug Research, International Center for Chemical and Biological Sciences, University of Karachi, Karachi, Pakistan; 3 Department of Pharmaceutical Chemistry, Faculty of Pharmacy, Anadolu University, Eskişehir, Turkey; 4 Department of Pharmacognosy, Faculty of Pharmacy, Anadolu University, Eskişehir, Turkey; 5 Biomedical Research Laboratory, College of Medicine, Alfaisal University, Riyadh, Saudi Arabia

**Keywords:** Alpha-glucosidase inhibitor, dithiocarbamate, piperazine, noncompetitive, allosteric

## Abstract

In continuation of our interest in identifying new α-glucosidase inhibitors with potential to become antidiabetic drugs, this work focuses on the study of 4-(dimethylaminoalkyl)piperazine-1-carbodithioate derivatives as α-glucosidase inhibitors. The eight heterocyclic piperazine-dithiocarbamate complexes studied in this work contain a variety of substitutions on their benzene ring exhibiting potent, noncompetitive inhibition of α-glucosidase. Dithiocarbamate and piperazine moieties are important pharmacophores with promising therapeutic prospects featuring facilitated drug delivery due to their lipophilic nature in addition to their α-glucosidase inhibitory activity. Enzyme kinetics, molecular dynamics simulations, and docking studies revealed that the target compounds bind to a new allosteric site that is located near the active site of α-glucosidase. Majority of molecular interactions of the compounds with the enzyme are mediated by hydrophobic contacts in addition to a number of important polar interactions. The current work identifies a number of chemical groups in the compounds that are responsible for potent inhibition of α-glucosidase. Moreover, it also provides new insights into understanding α-glucosidase inhibition by dithiocarbamate and piperazine-containing compounds that can be promising for development of new antidiabetic drugs.

## 1. Introduction

Intestinal α-glucosidases are a class of enzymes that are essential for digestion of dietary carbohydrates [1]. One of the treatment approaches for controlling hyperglycemia manifested in diabetes mellitus is inhibition of α-glucosidase [2]. The current therapeutic regime for treatment of diabetes includes only a limited number of oral α-glucosidase inhibitors namely acarbose, miglitol, and voglibose. Their low efficacy and gastrointestinal side effects are major obstacles to achieving promising therapeutic outcome. Unfortunately, no significant progress has been made in the drug development of oral α-glucosidase inhibitors. Therefore, this area needs attention to develop new α-glucosidase inhibitors with better efficacy, less side effects, and more therapeutic benefits [3–4].

Carbamate and piperazine moieties have been shown to exhibit promising biological activities for treatment of Alzheimer’s disease and a number of other central nervous system (CNS) disorders [5]. Dithiocarbamate, an isostere of carbamate, is a promising pharmacophore in the drug development of CNS-related disorders especially for drug delivery due to its lipophilic properties [6]. Moreover, the piperazine moiety exhibits diverse biological activities such as antiparasitic, antimicrobial, antipsychotic, antidepressant, and anxiolytic activities [7]. Furthermore, compounds bearing the piperazine moiety are also known to possess potent inhibitory activity against α-glucosidase including chiral piperazine diones [8], 2-furoic piperazides [9], triazole-containing piperazines [10], and phenylpiperazines [11]. Presently, there are a very limited number of reports on dithiocarbamates as inhibitors of α-glucosidase that include coumarin-containing dithiocarbamates [12], and benzyl carbamates [13]. Nevertheless, these derivatives are a promising class of emerging compounds that exhibit potent inhibition of α-glucosidase. Our recent work on dithiocarbamates α-glucosidase inhibitors targeted benzylamine and piperazine derivatives [14]. This work focuses on a series of novel 4-(dimethylaminoalkyl)piperazine-1-carbodithioate derivatives with various substitutions on their benzene ring exhibiting potent inhibition of α-glucosidase. The current work will not only provide new avenues to study the effects of the substitutions on the benzene ring but will also incorporate chemical diversity to dithiocarbamate inhibitors of α-glucosidase.

In this work, eight piperazine-containing carbodithioate derivatives containing various substitutions on their benzene ring (**PC1**–**PC8**) have been newly identified as noncompetitive inhibitors of α-glucosidase with focus on kinetics and computational studies. Docking and molecular dynamics (MD) simulations revealed important clues to understanding their interactions with the enzyme at molecular level. The work also identifies a new allosteric site on the enzyme to which the target compounds bind and influence its catalytic activity. Both dithiocarbamate and piperazine moieties along with the substitutions actively contribute to enzyme inhibition. MD simulations showed that the compounds bind to an allosteric site that is located near the active site revealing a number of important interactions with its amino acid residues. The current findings incorporate chemical diversity that would be valuable to understanding α-glucosidase inhibition by dithiocarbamate derivatives, and for searching new and effective α-glucosidase inhibitors as antidiabetic drugs.

## 2. Materials and methods

### 2.1. Synthesis and structure elucidation of the compounds

The design, synthesis, and structure elucidation of all target compounds studied in this work have been previously reported [15].

### 2.2. α-Glucosidase inhibition assay

All in vitro α-glucosidase inhibition assays were performed in a 96-well microplate format. The α-glucosidase inhibition assay reported elsewhere was customized to suit the experimental needs of the current work [16]. The assay mixture contained 50 mM sodium phosphate buffer (pH 6.8), 20 mU of α-glucosidase (EC 3.2.1.20) from *Saccharomyces cerevisiae* (Sigma Chemical Co., St. Louis, USA), and 1.0 mM of *p*-nitrophenyl-α-*D*-glucopyranoside (PNP-G) substrate. All target compounds were dissolved in DMSO with the assay mixture containing DMSO at a final concentration of 3%. All compounds were first incubated with the enzyme at 37 *°*C for 15 min followed by commencement of the enzyme reaction by adding the substrate. The reaction was continuously monitored at 400 nm in SpectraMax Plus 384^®^ microplate reader (Molecular Devices, CA, USA). The assay also included separate, parallel sets of control experiments containing no compounds as negative control, and acarbose as positive control. All assays were performed as four individual experiments out of which the values of mean and standard error of mean (SEM) were calculated.

### 2.3. α-Glucosidase inhibition kinetics

The enzyme kinetics assay procedure was essentially the same as mentioned above except that it was specifically designed for the kinetic work. The experiments included the addition of a range of target compound concentrations and that of the substrate (0.25–2.0 mM). In all experiments, only the linear portion of the enzyme reaction curves was selected for calculating initial velocities in the presence and absence of the target compounds. The type of inhibition was calculated from the Lineweaver-Burk plots which was further confirmed by the Dixon plot using Grafit 7.0.3 software (Erithacus Software Ltd., Staines, UK). The *K*_i_ values were also calculated from their respective Dixon plots. All kinetic experiments were conducted in quadruplicate from which mean *K*_i_ values and the SEM were calculated.

### 2.4. Computational studies

#### 2.4.1. α-glucosidase homology model building

The target compounds were docked into the homology model of *S. cerevisiae* α-glucosidase that we built and reported in our previous work [14].

#### 2.4.2. Docking and MD simulations

The chemical structures of the target compounds were drawn in the builder module in MOE-2015.1001 followed by addition of charges and energy minimization in MMFF94x force field [17]. A conformation search run was conducted in MOE to determine conformation with lowest energy which was selected for further studies. The default induced-fit docking protocol (triangle matcher) was chosen to perform molecular docking of the compounds that deployed two scoring functions namely London dG and GBVI/WSA dG. The protocol generated 30 poses for each compound followed by visual analysis of the top-ranked pose for each one.

In order to determine the conformational changes and stability of the predicted enzyme-ligand complexes, the docking poses were further analyzed by MD simulation. The AMBER18 suite was utilized for a production run of 50 ns simulation [18] for which all systems were prepared using the LEaP module in AMBER18. Application of parameters to the compounds and the enzyme was performed using AMBER force field (GAFF) and AMBERFF14SB, respectively. Assignment of the AM1-BCC charges to the ligands was carried out in the ANTECHAMBER program. Solvation of all systems was conducted using the cubic TIP3P water box leaving a margin of 10 Å under the periodic boundary conditions [19]. The systems were neutralized by replacing water molecules with appropriate counter ions. Initially, the energy minimization of each system was performed to remove the steric clashes between protein-ligand and protein-solvent molecules using the steepest descent procedure followed by conjugated gradient. Gradual heating was performed by raising the temperature from 0 K to 300 K for a time period of 500 ps. Each system was then equilibrated under the NPT ensemble for another 500 ps at a constant temperature (300 K) and pressure (P = 1 atm) with a coupling constant of 1.0 ps [20]. The SHAKE algorithm was utilized to constrain all hydrogen and other bonds [21]. The long-range electrostatic interactions were calculated using the particle mesh Ewald method with a distance cutoff of 8 Å [22]. A final production run of 50 ns for all systems was performed using the PMEMD.CUDA from AMBEER18 with an integration time of 2 fs that turned out trajectories which were analyzed in the CPPTRAJ module [23] of AMBER18.

#### 2.4.3. MM-PBSA / GBSA calculations

Calculation of free energy of binding is one of the promising approaches to computing ligand-protein binding affinities. For the current work, it was conducted by molecular mechanics-Poisson-Boltzmann surface area/generalized Born surface area (MM-PBSA / GBSA) method based on 1000 frames extracted from the equilibrated trajectories of all three systems. The Δ*G*_Binding_ between a ligand and a receptor to form a complex were calculated by using the following equation:


$$\begin{array}{l} \Delta {\text{G}}_{\text{Binding}}={\text{G}}_{\text{Complex}}-({\text{G}}_{\text{lig}}+{\text{G}}_{\text{rec}})\\ =\Delta {\text{E}}_{\text{MM}}+\Delta {\text{G}}_{\text{GB}}+\Delta {\text{G}}_{\text{SA}}-\text{T}\Delta \text{S}\\ =\Delta E_{\text{vdw}}+\Delta E_{\text{ele}}+\Delta {\text{G}}_{\text{GB}}+\Delta {\text{G}}_{\text{SA}}-\text{T}\Delta \text{S}, \end{array}$$

where the term Δ*E*_MM_ depicts the energy of gas-phase interaction between ligand and protein complex comprising the electrostatic energy contribution (Δ*E*_ele_) and van der Waals energy contribution (Δ*E*_vdw_). The Δ*G*_G_ and Δ*G*_SA_ represent the polar and nonpolar components of desolvation free energies, respectively. In this work, the ΔG_SA_ and ΔG_GB_ were estimated using the accessible surface area (SASA) model with the LCPO method: ΔGSA = 0.0072 × ΔSASA and generalized Born (GB) model, respectively.

## 3. Results and discussion

### 3.1. α-Glucosidase inhibition kinetics

**Figure 1:**
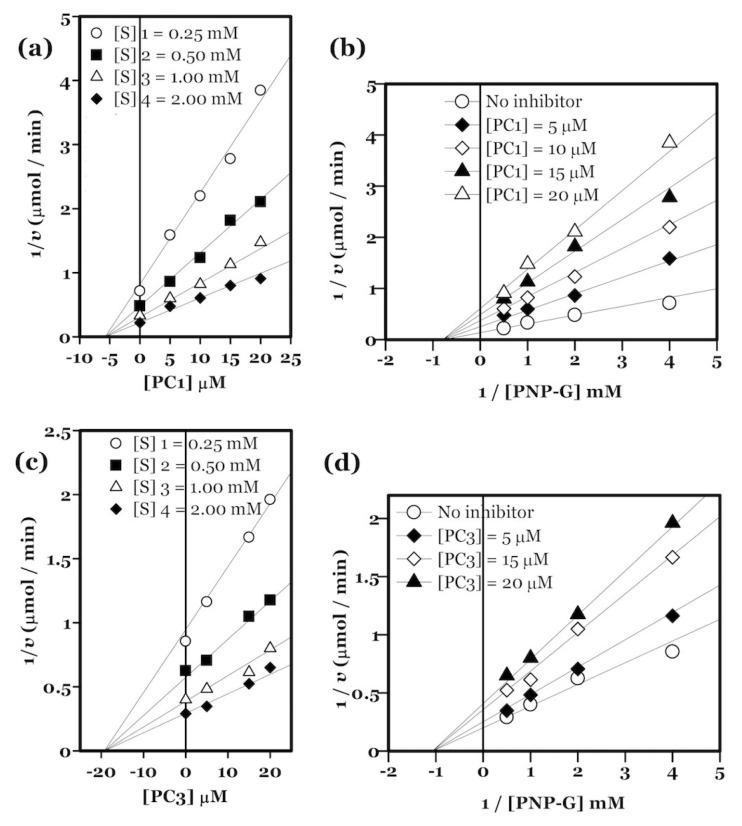
Representative Dixon (a,c) and Lineweaver–Burk plots (b,d) exhibiting noncompetitive inhibition by the compounds.

Compared to negative controls containing no inhibitors, a concentration-dependent decrease in the α-glucosidase reaction rate was observed in the presence of the target compounds. The *K*_i_ values of the compounds ranged from 5.75 to 29.36 μM as listed in Table 1. Analysis of the raw kinetic data from each experiment, with least standard of error, was conducted for determining the type of inhibition exhibited by each compound using the reciprocal transformation of the Michaelis-Menten equation. Lineweaver-Burk plots were used to screen the data for best fit into a potential type of inhibition [24]. Calculation of *V*_max_app and *K*_m_app values matching with the type of inhibition was also performed. Lineweaver–Burk plots defining the type of inhibition for each compound with least standard of error were considered for further analysis. All target compounds demonstrated noncompetitive inhibition of α-glucosidase as shown by their respective Lineweaver–Burk plots. Dixon plots {1/*v* versus [I]} [25] were utilized to determine the *K*_i_ values for all compounds by rearranging the Michaelis-Menten equation. The Dixon and Lineweaver–Burk plots for representative inhibitors are presented in Figure 1.

### 3.2. Structure-activity relationship

The target compounds possess substitutions on the phenyl ring adjacent to the oxoethyl group with varying number of alkyl carbon atoms connecting the piperazine-1-carbodithioate moiety. **PC1** (*K*_i_ = 5.75 μM) bearing a 4-chlorophenyl substitution, demonstrated most potent inhibition of α-glucosidase. **PC2** (*K*_i_ = 12.70 μM), derivatized by a 2,4-dichlorophenyl group, showed 2-fold less activity than **PC1**. In addition to these substitutions in **PC1** and **PC2**, the differences in their potency also appear to be due to the oxobutyl and oxoethyl groups, respectively. **PC1** is extended by one more carbon atom, which is apparently an important feature contributing to more potent inhibition of the enzyme than by **PC2**. This feature is also present in **PC6** (*K*_i_ = 20.93 μM); however, its phenyl ring is unsubstituted, exhibiting much less potency when compared to **PC1**, emphasizing the importance of the phenyl ring substitutions for potent inhibition of the enzyme. The chlorine-containing compounds (**PC1** and **PC2**) were identified as relatively more potent inhibitors than the ones bearing other groups. The *K*_i_ values of the rest of the compounds, carrying fluorine, bromine and hydroxyl groups, were found to be similar to each other.

**PC3, PC4**, and **PC5** exhibited similar inhibitory activities despite having differences in the position of their substituent groups. All of these compounds bear a 4-fluorophenyl substitution except for **PC3** which bears an additional fluorine atom at the 2-position of the phenyl ring (2,4-difluorophenyl substitution). Moreover, **PC4** is slightly extended due to its oxopropyl group compared to **PC3** and **PC5** that carry an oxoethyl group. All these differences in the types and positions of the substituent groups did not affect the activities of these compounds. It is interesting to note that bromine is not optimal for enzyme inhibition; **PC8** bearing 4-bromophenyl and oxoethyl groups showed least activity (*K*_i_ = 29.36 μM). It is clear from the above data that chlorine substantially contributes to potent inhibition of α-glucosidase. However, comparison of other halogen-containing compounds (**PC3**, **PC4**, **PC5** and **PC8**) with the ones devoid of halogens such as **PC6** and **PC7** (bearing hydroxyl groups) revealed no major differences in their activities. **PC9** possessing an unsubstituted phenyl ring and an oxopropyl group showed no inhibitory activity, which is very similar to **PC6** carrying an oxobutyl group but with one more carbon atom connecting the two moieties. Interestingly, a single carbon atom makes a striking difference between these two compounds rendering one to be inactive. This is consistent with the results discussed above showing that oxobutyl moiety partly contributes to potent α-glucosidase inhibition in addition to phenyl substitutions. Moreover, the additional carbon atom in **PC6** extends the molecule that appears to facilitate access to enzyme residues for a better interaction.

### 3.3. Computational studies

As mentioned earlier, all target compounds noncompetitively inhibited α-glucosidase which infers that they bind to a site on the enzyme other than the active site. This is consistent with the previous studies by Proenca et al. [26] who reported that the larger size of the binding cavity of α-glucosidase is primarily responsible for a range of inhibitor binding modes leading to various types of enzyme inhibition. In order to cater to search for a potential binding site, blind docking protocols were followed for the compounds along with their binding modes. The docking results identified a new allosteric site that is located in the close proximity of the active site. The site is primarily composed of Lys152, Ser153, Phe154, His236, Asn238, Val300, Glu301, Gly303, Pro306, Phe307, Arg309, and Arg353 residues. A cluster of compounds with various conformations was found at this site where the compounds specifically interact with Lys152, Phe154, Val300, Pro306, Phe307, and Arg309 residues. The target compounds (**PC1, PC2**, and **PC5**) selected for docking and MD simulations bind to this region of the enzyme where their molecular interactions take place with various residues mentioned above. The docking scores for the compounds are listed in Table 2.

We compared our newly-identified allosteric site with known ones reported in the literature for yeast α-glucosidase [27–29], and with the one identified in our previous work [14]. Majority of the reported sites are located in the vicinity of each other as revealed by their amino acid sequence. Comparison of their sequence with that of our site indicated that it is located in the close proximity to at least three of the known sites [27–28] including that of our previously identified site [14]. This shows that the site is part of a large enzyme region where those known sites are generally located. Despite this close proximity, our site is formed of amino acid residues that are different from that of the known ones; hence, the site is new which is constituted by unique amino acid residues.

Since the target compounds are noncompetitive inhibitors of α-glucosidase, they apparently regulate substrate binding through the allosteric site. We have previously shown that all known allosteric sites are located in close proximity to the active site [14] that appears to directly influence the binding of the substrate (PNP-G) by apparently inducing conformational changes in the active site pocket resulting in enzyme inhibition. Study of the exact mechanism of the conformational changes warrants separate future investigations involving dedicated and exhaustive computational work.

Since molecular docking only depicted a static picture of the molecular interactions of the ligands with the enzyme, we further expanded our work by performing MD simulation experiments that highlighted various system properties with respect to time that were difficult to attain otherwise.

### 3.4. Structural stability analysis

**Figure 2:**
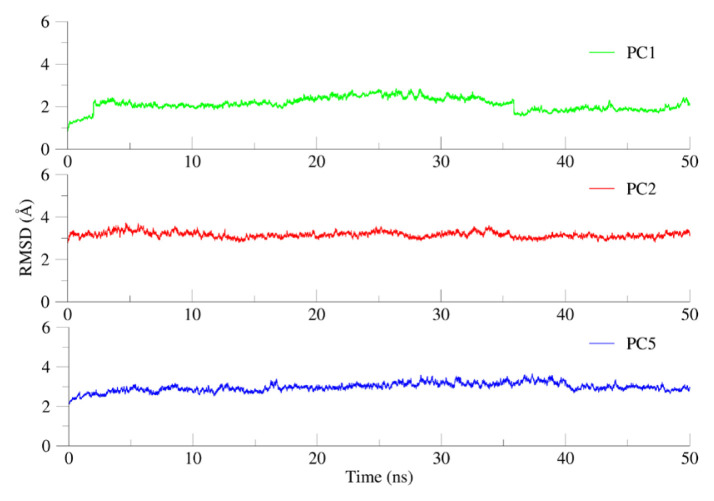
Root mean square deviation (RMSD) as a function of time for the protein backbone. A production run of 50 ns revealed that all systems demonstrated variable deviations with an average RMSD value of 2.5–3.2 Å with stable internal motion.

Root mean square deviation (RMSD) plays a crucial role in describing the system stability during MD simulations. Initially, the stability and deviation of each complex was investigated in order to calculate the positional differences of backbone atoms from its initial structure. RMSD calculations (Figure 2) revealed that the assembly was not fully equilibrated until 10 ns into the production run (as determined by the protein RMSD); therefore, only the remaining 40 ns were considered for subsequent calculations. As evident from Figure 2, all systems displayed variable deviations with an average RMSD value of 2.5–3.2 *Å with stable internal motion.*

**Figure 3:**
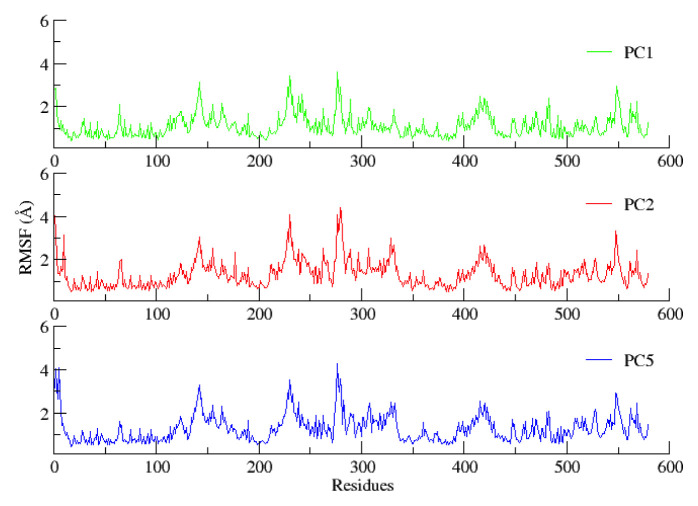
Root mean square fluctuation (RMSF) as a function of time for the protein backbone.

Root mean square fluctuation (RMSF) analysis was performed to investigate the flexibility of α-glucosidase residues. The results over the time scale for all three systems are presented in Figure 3. A large fluctuation in residues 229, 278, and 400–435 was observed for all three enzyme-inhibitor complexes. Visual assessment of these residues interpreted by the secondary structure suggested that these residues are part of the loops which are highly flexible in nature. Apart from this fluctuation, other sites were calculated to be comparatively stable. Analysis of the MD trajectories revealed that all the target compounds (**PC1, PC2**, and **PC5**) presented significant dislocation in the coordinates before and after simulation, exhibiting differences of 2.06, 2.48, and 2.54 *Å, respectively. However, the interactions were later stabilized by hydrophobic interactions displayed by the compounds*.

### 3.5. Molecular interactions

**Figure 4:**
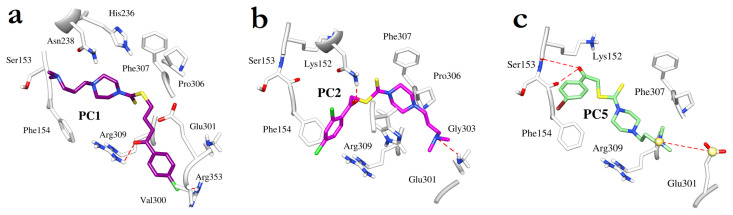
Binding modes of (a) **PC1**, (b) **PC2**, and (c) **PC5**. The enzyme residues are drawn as thick white sticks, and the dashed red lines represent hydrogen bonding interactions. For clarity purposes, residues only interacting with the inhibitors are shown.

Time-dependent analysis of **PC1** bound to the enzyme revealed that its carbonyl group is involved in hydrogen bonding interaction with the side chain of Arg309 at a distance of 2.09 Å. The complex is further stabilized by the halogen bonds between the C_l_ at *para* position and Arg353, and Gln347 residues through chlorine. The carbodithioate moiety of **PC1** is involved in π-sulfur interactions with His236, whereas the aliphatic chain of its dimethylaminopropyl group forms hydrophobic contacts with Asn238 and Phe154 residues (Figure 4a). Its piperazine moiety extends and stabilizes the attached aliphatic chain that is aligned between the Asn238 and Phe154 residues. Detailed interaction profiles of selected target compounds with important residues of the binding site are provided in Table 3.

The carbonyl group of **PC2** (Figure 4b) is involved in hydrophilic interactions with the side chain of Asn238 at a distance of 3.14 Å. Its carbodithioate moiety is stabilized by His276 residue through hydrophobic interactions. The carbonyl group of **PC5** is stabilized by hydrophilic interactions with the side chain of Ser153 at a distance of 2.56 Å (Figure 4c), while its carbodithioate moiety forms hydrophobic contacts with Phe154 and Arg309 residues. The dichlorobenzene ring of **PC2** and the fluorobenzene ring of **PC5** are involved in parallel π-stacking interactions with Phe154 residue. Additionally, **PC2** is involved in hydrogen bonding interaction with Gly303 whereas **PC5** is further stabilized by a salt bridge between its tertamine and the carboxylate group of Glu301 residue. These interactions exhibited by both compounds could not have been possible without assistance from the piperazine ring and the aliphatic chain that extend the molecule thus enabling their access to respective residues. The results indicate that hydrophobic interactions have a key influence on enzyme-inhibitor binding as expected due to the lipophilic characteristics of the compounds. However, there is also a significant role of the carbonyl groups of the compounds in enzyme inhibition that establish polar contacts with the residues located in the allosteric site.

### 3.6. Analysis of free energy of binding

The MM-PBSA/GBSA free energy calculations were utilized to obtain insights into the forces involved in the enzyme-inhibitor binding. To determine the binding affinities of all three systems, 1000 frames were extracted from the last 5 ns of the MD production. The calculated binding energy for **PC1, PC2**, and **PC5** were −37.0360, −38.5350, and −35.3002, respectively. In order to comprehend the influence of specific energy terms in the binding process, the total free energy was disintegrated into Eele, Evdw, ESURF, and EGB energy components. The results presented in Table 4 suggested that the intermolecular van der Waals interactions (Δ*E*_int vdW_) contribute more significantly to the binding free energies than electrostatic interactions (Δ*G*_ele_), which is consistent with the MD results and with the chemical nature of the compounds. The nonpolar solvation terms, corresponding to burial SASA upon inhibitor binding, favorably contributed to the binding affinity to some extent. However, the total binding affinity appears to be due to a more complex interplay between all these components.

## 4. Conclusion

The novel 4-(dimethylaminoalkyl)piperazine-1-carbodithioate derivatives studied in this work demonstrate noncompetitive inhibition of yeast α-glucosidase. The compounds feature various halogen substitutions along with the oxoalkyl groups of different carbon lengths that exerted cumulative effect on enzyme inhibition. The results showed that the halogen substitutions on the benzene ring and the length of the oxoalkyl groups were crucial for α-glucosidase inhibition. Additionally, the docking and MD studies have helped to identify a new allosteric site on the enzyme to which the target compounds bind and apparently influence the catalytic activity. Computational studies have predicted interactions of the target compounds with the enzyme at molecular level that is predominantly governed by hydrophobic and to some degree by electrostatic interactions. The piperazine moiety allows extension of the aliphatic chain and the dimethylaminopropyl group for access to the region of the allosteric site where they form hydrophobic and polar contacts with the residues, respectively. The current work reveals clues to understanding α-glucosidase inhibition by piperazine-containing dithiocarbamate derivatives at molecular level that will be useful for new antidiabetic drug discovery.

## Figures and Tables

**Table 1:** The structures and *K*_i_ values of the target inhibitors, all of which noncompetitively inhibited α-glucosidase. The *K*_i_ values are the mean of quadruplet experiments with SEM.

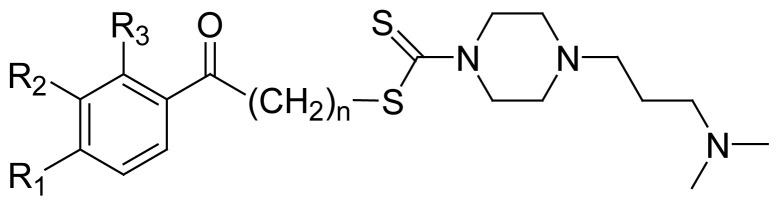					
No.	R_1_	R_2_	R_3_	n	*K*_i_ (μM ± SEM)
**PC1**	Cl	H	H	3	5.75 ± 0.20
**PC2**	Cl	H	Cl	1	12.70 ± 0.34
**PC3**	F	H	F	1	19.25 ± 0.51
**PC4**	F	H	H	2	19.38 ± 0.42
**PC5**	F	H	H	1	19.82 ± 0.51
**PC6**	H	H	H	3	20.93 ± 0.28
**PC7**	OH	OH	H	1	21.28 ± 0.51
**PC8**	Br	H	H	1	29.36 ± 0.60
**PC9**	H	H	H	2	Inactive
Acarbose					4.73 ± 0.33

**Table 2:** Docking scores for **PC1**, **PC2**, and **PC5**.

Inhibitor	Docking scores
**PC1**	−7.2339
**PC2**	−7.9760
**PC5**	−8.2346
**PNP-G**	−7.0442

**Table 3:** The ligand–protein interaction profiles of selected inhibitors within the α-glucosidase binding site (abbreviations: MD = molecular dynamics; Lig. = ligand).

Inhibitor	van der Waals Interactions post-MD	Hydrogen bond and salt bridge interactions post-MD		
		Acceptor atom	Donor atom	Distance Å
**PC1**	Ser153, Phe154, Val300, Phe307, Glu301, Arg353	Lig: Carbonyl=O9 Lig: Cl	Arg309-NH1 Arg353-NH1	2.9 ± 0.36 3.34 ± 0.52
**PC2**	Lys152, Ser153, Phe154, Glu301, Pro306, Phe307	Lig: Carbonyl=O Lig: N2	Asn238-NH2 Gly303-NH1	2.04 ± 0.44 2.3 ± 0.62
**PC5**	Lys152, Ser153, Phe154, Glu301, Phe307, Arg309	Lig: N2	Glu301-OE	3.9 ± 0.58

**Table 4:** Contribution of each energy component to the binding of all three simulated systems (kcal/mol).

System	E_vdw_	E_ele_	E_GB_	ESURF	***ΔG***_binding_ (GB)
**PC1**	−44.5426	−20.0809	30.4908	−4.4710	−38.0360 *± 2.2*
**PC2**	−46.3578	−18.4149	31.7248	−5.4826	−36.5350 ± 2.0
**PC5**	−49.8309	−18.0814	38.2362	−5.6240	−35.3002 ± 2.4

## References

[1] Ernst HA Lo Leggio L Willemoës M Leonard G Blum P Larsen S Structure of the Sulfolobus solfataricus α-glucosidase: implications for domain conservation and substrate recognition in GH3 Journal of Molecular Biology 2006 358 1106 1124 16580018 10.1016/j.jmb.2006.02.056

[2] Maki KC Carson ML Miller MP Turowski M Bell M Wilder DM Reeves MS High-viscosity hydroxypropylmethylcellulose blunts postprandial glucose and insulin responses Diabetes Care 2007 30 1039 1043 17259476 10.2337/dc06-2344

[3] Ghani U Alpha-glucosidase inhibitors: clinically promising candidates for antidiabetic drug discovery Oxford, UK Elsevier 2020

[4] Ghani U Re-exploring promising α-glucosidase inhibitors for potential development into oral anti-diabetic drugs: Finding needle in the haystack European Journal of Medicinal Chemistry 2015 103 133 162 10.1016/j.ejmech.2015.08.043 26344912

[5] Asadipour A Alipour M Jafari M Khoobi M Emami S Novel coumarin-3-carboxamides bearing N-benzylpiperidine moiety as potent acetylcholinesterase inhibitors European Journal of Medicinal Chemistry 2013 70 623 630 24211638 10.1016/j.ejmech.2013.10.024

[6] Wang XJ Xu HW Guo LL Zheng JX Xu B Guo X Zheng CX Liu HM Synthesis and in vitro antitumor activity of new butenolide-containing dithiocarbamates Bioorganic & Medicinal Chemistry Letters 2011 21 3074 3077 21486694 10.1016/j.bmcl.2011.03.029

[7] Brito AF Moreira LKS Menegatti R Costa EA Piperazine derivatives with central pharmacological activity used as therapeutic tools Fundamentals of Clinical Pharmacology 2019 33 13 24 10.1111/fcp.12408 30151922

[8] Arcelli A Balducci D Porzi G Sandri M Chiral piperazine-2,5-dione derivatives as effective alpha-glucosidase inhibitors. Part 4 Chemical Biodiversity 2010 7 225 228 10.1002/cbdv.200900096 20087993

[9] Abbasi MA Hassan M Ur-Rehman A Siddiqui SZ Hussain G Shah SAA Ashraf M Shahid M Seo SY 2-Furoic piperazide derivatives as promising drug candidates of type 2 diabetes and Alzheimer’s diseases: In vitro and in silico studies Computational Biology & Chemistry 2018 77 72 86 10.1016/j.compbiolchem.2018.09.007 30245349

[10] Mermer A Demirbas N Demirbas A Colak N Ayaz FA Alagumuthu M Arumugam S Synthesis, biological activity and structure activity relationship studies of novel conazole analogues via conventional, microwave and ultrasound mediated techniques Bioorganic Chemistry 2018 81 55 70 10.1016/j.bioorg.2018.07.036 30118986

[11] Abbasi MA Anwar A Rehman A Siddiqui SZ Rubab K Synthesis, enzyme inhibition and molecular docking studies of 1-Arylsulfonyl-4-phenylpiperazine derivatives Pak Journal of Pharmaceutical Sciences 2017 30 1715 1724 29084694

[12] Mollazadeh M Mohammadi-Khanaposhtani M Valizadeh Y Zonouzi A Faramarzi MA Novel coumarin containing dithiocarbamate derivatives as potent α-glucosidase inhibitors for management of type 2 diabetes Medicinal Chemistry 2020 17 264 272 10.2174/1573406416666200826101205 32851964

[13] Popović-Djordjević JB Jevtić II Grozdanić NDJ Šegan SB Zlatovićet MV α-Glucosidase inhibitory activity and cytotoxic effects of some cyclic urea and carbamate derivatives Journal of Enzyme Inhibition and Medicinal Chemistry 2017 32 298 303 10.1080/14756366.2016.1250754 28100083 PMC6010093

[14] Ghani U Ashraf S Ul-Haq Z Mujamammi AH Özkay Y Demirci F Kaplancikli ZA Dithiocarbamate derivatives inhibit α-glucosidase through an apparent allosteric site on the enzyme Chemical Biology & Drug Design 2021 98 283 294 10.1111/cbdd.13897 34047492

[15] Acar Cevik UA Levent S Saglık BN Ozkay Y Kaplancıklı ZA Synthesis of novel 4-(dimethylaminoalkyl)piperazine-1-carbodithioate derivatives as cholinesterase inhibitors Letters in Drug Design and Discovery 2017 14 528 539 10.2174/1570180813666160923105636

[16] Ma K Han J Bao L Wei T Liu H Two sarcoviolins with antioxidative and α-glucosidase inhibitory activity from the edible mushroom *Sarcodon leucopus* collected in Tibet Journal of Natural Products 2014 77 942 947 24645629 10.1021/np401026b

[17] Molecular Operating Environment (MOE) 2019010, Chemical Computing Group ULC 1010 Sherbooke St. West, Suite #910, Montreal, QC, Canada, H3A 2R7 2018

[18] Lee TS Cerutti DS Mermelstein D Lin C LeGrandet S GPU-accelerated molecular dynamics and free energy methods in Amber18: performance enhancements and new features Journal of Chemical Information & Modeling 2018 58 2043 50 30199633 10.1021/acs.jcim.8b00462 PMC6226240

[19] Jorgensen WL Chandrasekhar J Madura JD Impey RW Klein ML Comparison of simple potential functions for simulating liquid water Journal of Computational Physics 1983 79 926 35

[20] Andersen HC Molecular dynamics simulations at constant pressure and/or temperature Journal of Computational Physics 1980 72 2384 93

[21] Ryckaert JP Ciccotti G Berendsen HJ Numerical integration of the cartesian equations of motion of a system with constraints: molecular dynamics of n-alkanes Journal of Computational Physics 1977 23 327 41

[22] Cheatham TI Miller JL Fox T Darden TA Kollman PA Molecular dynamics simulations on solvated biomolecular systems: the particle mesh Ewald method leads to stable trajectories of DNA, RNA, and proteins Journal of American Chemical Society 1995 117 4193 4194

[23] Roe DR Cheatham III PTRAJ TE CPPTRAJ: software for processing and analysis of molecular dynamics trajectory data Journal of Chemical Theory & Computation 2013 9 3084 95 26583988 10.1021/ct400341p

[24] Lineweaver H Burk D The determination of enzyme dissociation constants Journal of American Chemical Society 1934 56 658 666

[25] Dixon M The determination of enzyme inhibitor constants Biochemical Journal 1953 55 170 171 13093635 10.1042/bj0550170 PMC1269152

[26] Proença C Freitas M Ribeiro D Oliveira EF Sousa JL Tomé SM Ramos MJ Silva AM Fernandes PA Fernandes E α-Glucosidase inhibition by flavonoids: an in vitro and in silico structure–activity relationship study Journal of Enzyme Inhibition and Medicinal Chemistry 2017 32 1216 1228 28933564 10.1080/14756366.2017.1368503 PMC6009965

[27] Liu Y Ma L Chen WH Park H Ke Z Wang B Binding mechanism and synergetic effects of xanthone derivatives as non-competitive α-glucosidase inhibitors: A theoretical and experimental study The Journal of Physical Chemistry B 2013 117 13464 13471 24083955 10.1021/jp4067235

[28] Brindis F Rodríguez R Bye R González-Andrade M Mata R (Z)-3-butylidenephthalide from Ligusticum porteri, an α-glucosidase inhibitor Journal of Natural Products 2011 74 314 320 20879744 10.1021/np100447a

[29] Ding H Hu X Xu X Zhang G Gong D Inhibitory mechanism of two allosteric inhibitors, oleanolic acid and ursolic acid on α-glucosidase International Journal of Biological Macromolecules 2018 107 1844 1855 10.1016/j.ijbiomac.2017.10.040 29030193

